# Association of genetic variants related to combined lipid-lowering and antihypertensive therapies with risk of cardiovascular disease: 2 × 2 factorial Mendelian randomization analyses

**DOI:** 10.1186/s12916-024-03407-x

**Published:** 2024-05-20

**Authors:** Ying Li, Hongwei Liu, Chong Shen, Jianxin Li, Fangchao Liu, Keyong Huang, Dongfeng Gu, Yun Li, Xiangfeng Lu

**Affiliations:** 1 Department of Epidemiology, Center for Global Health, School of Public Health, Nanjing Medical University, Nanjing, 211166 China; 2 Department of Epidemiology, Fuwai Hospital, National Center for Cardiovascular Diseases, Chinese Academy of Medical Sciences and Peking Union Medical College, Beijing, 100037 China; 3 Key Laboratory of Cardiovascular Epidemiology, Chinese Academy of Medical Sciences, Beijing, 100037 China; 4 Department of Cancer Epidemiology, The Affiliated Cancer Hospital of Zhengzhou University and Henan Cancer Hospital, Zhengzhou, 450008 China; 5 Research Units of Cohort Study On Cardiovascular Diseases and Cancers, Chinese Academy of Medical Sciences, Beijing, 100730 China; 6 School of Public Health and Emergency Management, Southern University of Science and Technology, Shenzhen, 518055 China; 7 School of Medicine, Southern University of Science and Technology, Shenzhen, 518055 China; 8 School of Public Health, North China University of Science and Technology, Tangshan, 063210 China

**Keywords:** Lipid-lowering drugs, Antihypertensive drugs, Factorial Mendelian randomization, Cardiovascular disease

## Abstract

**Background:**

Lipid-lowering drugs and antihypertensive drugs are commonly combined for cardiovascular disease (CVD). However, the relationship of combined medications with CVD remains controversial. We aimed to explore the associations of genetically proxied medications of lipid-lowering and antihypertensive drugs, either alone or both, with risk of CVD, other clinical and safety outcomes.

**Methods:**

We divided 423,821 individuals in the UK Biobank into 4 groups via median genetic scores for targets of lipid-lowering drugs and antihypertensive drugs: lower low-density lipoprotein cholesterol (LDL-C) mediated by targets of statins or proprotein convertase subtilisin/kexin type 9 (PCSK9) inhibitors, lower systolic blood pressure (SBP) mediated by targets of β-blockers (BBs) or calcium channel blockers (CCBs), combined genetically lower LDL-C and SBP, and reference (genetically both higher LDL-C and SBP). Associations with risk of CVD and other clinical outcomes were explored among each group in factorial Mendelian randomization.

**Results:**

Independent and additive effects were observed between genetically proxied medications of lipid-lowering and antihypertensive drugs with CVD (including coronary artery disease, stroke, and peripheral artery diseases) and other clinical outcomes (ischemic stroke, hemorrhagic stroke, heart failure, diabetes mellitus, chronic kidney disease, and dementia) (*P* > 0.05 for interaction in all outcomes). Take the effect of PCSK9 inhibitors and BBs on CVD for instance: compared with the reference, PCSK9 group had a 4% lower risk of CVD (odds ratio [OR], 0.96; 95%CI, 0.94–0.99), and a 3% lower risk was observed in BBs group (OR, 0.97; 95%CI, 0.94–0.99), while combined both were associated with a 6% additively lower risk (OR, 0.94; 95%CI, 0.92–0.97;* P* = 0.87 for interaction).

**Conclusions:**

Genetically proxied medications of combined lipid-lowering and antihypertensive drugs have an independent and additive effects on CVD, other clinical and safety outcomes, with implications for CVD clinical practice, subsequent trials as well as drug development of polypills.

**Supplementary Information:**

The online version contains supplementary material available at 10.1186/s12916-024-03407-x.

## Background

Dyslipidemia and hypertension are the established and major risk factors for cardiovascular disease (CVD) and they often coexist [1–4]. Numerous randomized controlled trials (RCT) and meta-analyses have demonstrated the great cardiovascular benefits of lowering blood lipids or blood pressure [1, 5–7]. Because the biological and clinical effects of combining both antihypertension and lowering blood lipids may be accumulative in a certain way [8, 9], combined treatment may benefit the population to a greater extent. Therefore, it is crucial to explore whether there exist interactions and quantify the effects of the combined treatment with lipid-lowering drugs and antihypertensive drugs on cardiovascular risk.

However, controversies existed among the RCT regarding the effects of combined lipid-lowering and antihypertensive drugs on CVD. The Anglo-Scandinavian Cardiac Outcomes Trial and a systematic review suggested synergistic effects on cardiovascular outcomes between statins and the antihypertensive drugs [10, 11]. But a 2 × 2 factorial RCT evaluated the effects of combined simvastatin and enalapril on CVD and suggested no interaction between them [12]. And another similar RCT including 12,705 participants observed no significant interaction between rosuvastatin for lowering blood lipids and candesartan plus hydrochlorothiazide for antihypertension on cardiovascular events [13]. Ference et al. conducted a factorial Mendelian randomization (MR) and found no synergy of lower low-density lipoprotein cholesterol (LDL-C) and systolic blood pressure (SBP) with CVD risk [14]. Additionally, in an experiment on mice, no synergistic effect was seen for the cotreatment of amlodipine and atorvastatin [15, 16]. Regarding other clinical and safety outcomes related to lipid-lowering drugs or antihypertensive drugs, it is unclear whether there exist interactions between lipid-lowering drugs and antihypertensive drugs on clinical adverse events (e.g., diabetes mellitus (DM) and dementia). In addition, these current factorial clinical trials regarding cardiovascular disease mainly focused on statin but not proprotein convertase subtilisin/kexin type 9 (PCSK9) inhibitors for lipid lowering, and β-blockers (BBs) for antihypertension were rarely studied. The biological effects on CVD risk for these medications may be different due to specific mechanism. Therefore, it is imperative to comprehensively evaluate the interactions between the commonly used lipid-lowering and antihypertensive drugs [17–19].

Using 2 × 2 factorial MR analyses could explore these issues efficiently and conveniently. Factorial MR study is based on the principle of MR, analogous to 2 × 2 factorial randomized controlled trial. This approach is frequently used to estimate causal effect interactions between exposures or identify the interactions between interventions using genetic variants as proxies for specific treatment to naturally randomize participants into 2 × 2 groups [20]. It can provide reliable genetic evidence before investing in the RCT which is time-consuming and relatively difficult to conduct. Currently, several commonly used lipid-lowering and antihypertensive drugs have appropriate genetic instruments as proxies to facilitate the analyses.

In this study, based on the large-scale study population in UK Biobank, we conducted 2×2 factorial MR analyses to evaluate the interactions between different lipid-lowering drugs (statins and PCSK9 inhibitors) and antihypertensive drugs (BBs and calcium channel blockers [CCBs]) on risk of CVD (defined as a composite of CAD, stroke and peripheral artery diseases [PAD]), other clinical and safety outcomes (ischemic stroke [IS], hemorrhagic stroke [HS], heart failure [HF], DM, chronic kidney disease [CKD], and dementia).

## Methods

### Study population

The UK Biobank recruited around 500,000 participants (aged 37–73) from 2006 to 2010 across 22 assessment centers in England, Scotland, and Wales. Participants completed a series of physical examinations and baseline questionnaires, such as socio-demographic characteristic, lifestyle, and self-reported health conditions [21]. Participants also provided biological samples including blood at baseline for biochemical assays and genotyping. We restricted the analyses to unrelated individuals who were third degree or less related to each other and identified as the White of European ancestry based on self-report and genetic profiling. And we excluded participants with mismatched information between self-reported and genetic sex and who has withdrawn. This process was shown in detail in the flow chart (Additional file 2: Fig. S1).

### Exposures and genetic instruments

The genetic instruments for proxies of statin and PCSK9 inhibitors were obtained from the publicly available summary data of a genome-wide association study (GWAS) meta-analysis of LDL-C levels in the Global Lipids Genetics Consortium [22]. Genetic variants were identified within 100 kb on either side of the target gene (*HMGCR* for statins and *PCSK9* for PCSK9 inhibitors) and associated with LDL-C at genome-wide significance level (*P* < 5.0 × 10^–8^) and were permitted to be in weak linkage disequilibrium (*r*^2^ < 0.20) with each other to increase the proportion of variance in each respective drug target explained by the instruments, maximizing the instrument strength [23].

For the genetic instruments of antihypertensive drugs, we used genetic proxies for CCBs and BBs provided by previous studies [24]. The genes encoding pharmacological targets for the antihypertensive drugs in DrugBank (*CACNA1D*, *CACNA1F*, *CACNA2D1*, *CACNA2D2*, *CACNA1S*, *CACNB1*, *CACNB2*, *CACNB3*, *CACNB4*, *CACNG1*, and *CACNA1C* for CCBs, *ADRB1* for BBs) were identified [24]. Genetic variants in these genes or regulatory gene regions and associated with SBP in GWAS meta-analysis of UK Biobank and International Consortium of Blood Pressure were selected as genetic instruments of the antihypertensive drugs. The GWAS meta-analysis was performed in up to 757,601 participants, with adjustment for age, age^2^, sex, body mass index (BMI), and study-specific covariates [25]. The genetic proxies and the effect sizes of which were shown in Additional file 1: Table S1. Regarding other clinical drugs like angiotensin-converting enzyme inhibitors (ACEI), there were not enough genetic variants (only 1 single-nucleotide polymorphism [SNP]) identified in ACE gene encoding pharmacological targets [24, 26], leading to low power and the failure of naturally random allocation.

We constructed the genetic scores for statin, PCSK9 inhibitors, BBs, and CCBs by combining all variants selected for each drug to create an instrument that could overcome weak effect of single variant and that would allow us to randomly allocate the participants into approximately equal-sized groups to perform the 2 × 2 factorial analyses. The genetic scores for these four drugs were constructed using the selected instrumental variables, with the effect size of each genetic variant as the weight. And the genetic scores below the median represented genetically proxied medications of lipid-lowering or antihypertensive drugs, which were related to lower LDL-C or SBP.

In our study, we used genetic scores as genetic proxies for exposures to lipid-lowering and antihypertensive drugs. These scores have direct effects on low-density lipoprotein-cholesterol (LDL-C) and systolic blood pressure (SBP). SBP (UK Biobank fields 4080) was measured twice at baseline, following the standard protocol [27]. LDL-C (UK Biobank fields 30780), as part of the UKB Biomarker project, was determined by biochemical arrays and analyzed by enzymatic selective protection [28]. As for the individuals who were taking antihypertensive drugs (UK Biobank fields 6153 and 6177), blood pressure was adjusted by adding 15 mmHg for SBP [25]. For those on lipid-lowering medication (UK Biobank fields 6153 and 6177), we replaced their LDL-C values by LDL-C/0.7 [29].

### Outcomes

The primary outcome of our study was CVD, defined as a composite of CAD, PAD and stroke. In addition, we analyzed other clinical and safety outcomes, including IS, HS, HF, DM, CKD, and dementia. These outcomes were identified using International Classification of Disease (ICD) 9 and 10 codes, non-cancer illness code (self-reported), as well as Classification of Interventions and Procedures. Detailed definitions of these outcomes were elaborated in Additional file 1: Table S2.

### Statistical analyses

The application of MR must satisfy three core assumptions, including (1) the genetic instrumental variables are strongly associated with the exposure of interest (relevance assumption); (2) they are not associated with the outcome via a confounding pathway (independence assumption); and (3) the instrument variables do not affect the outcome directly, only possibly indirectly via the exposure (exclusion restriction assumption). For the relevance assumption, we evaluated the association of each drug score with LDL-C or SBP and all outcomes, using logistic or linear regression adjusted for age, sex, assessment center, SNP array, and top ten genetic principal components. And we estimated the proportion of variance for each drug explained by the instrument (*R*^2^) and *F* statistics. As a convention, an *F* statistic of at least ten indicates no weak instrument bias. To assess independence assumption, we investigated the associations of drugs scores with potential confounders (BMI, smoking, and drinking) and further adjusted the factors with significant associations (*P* < 0.05) in the sensitivity analyses. Regarding the exclusion restriction assumption, it is somewhat more difficult to demonstrate the validity. We assessed pleiotropy using the method of MR-Egger regression by conducting two sample MR with GWAS summary data.

**Fig. 1:**
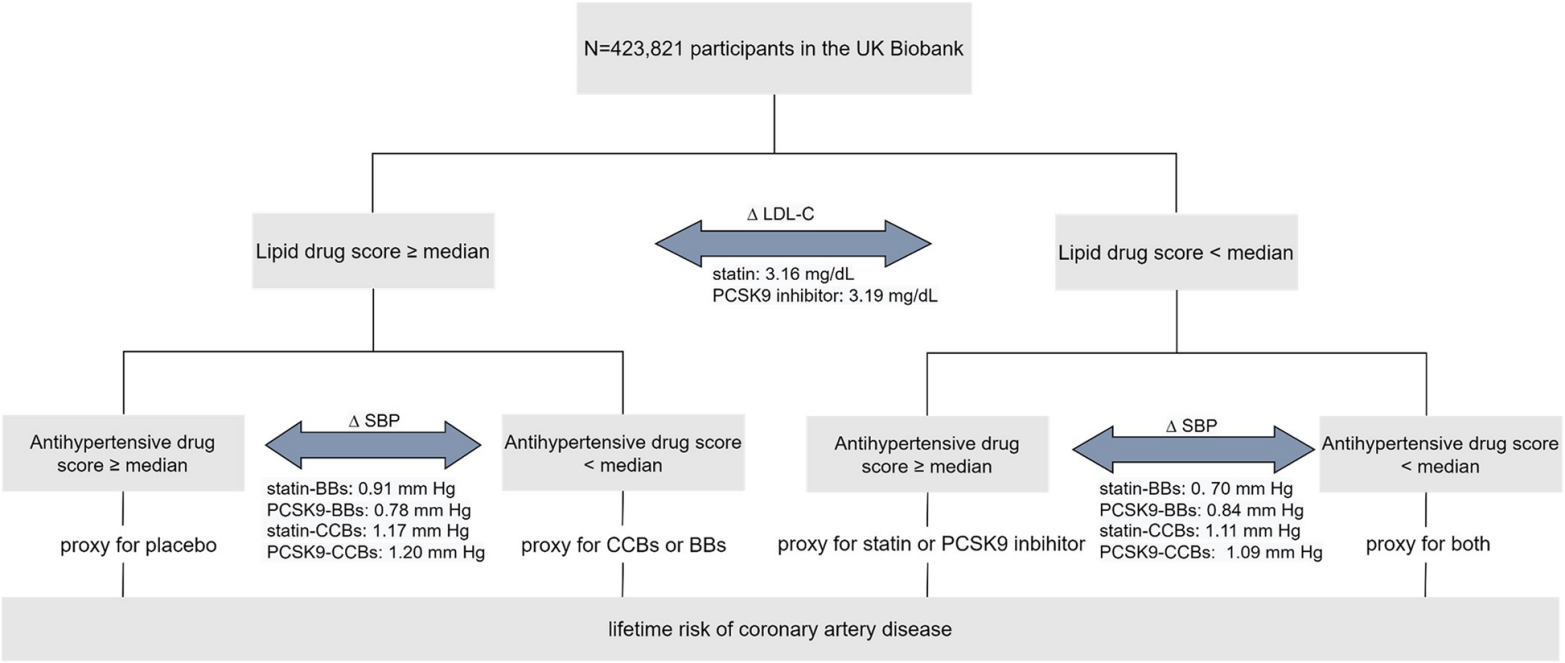
Study design of the 2 × 2 factorial Mendelian randomization analysis in the UK Biobank. BBs beta-blockers, CCBs calcium channel blockers, SBP systolic blood pressure, LDL-C low-density lipoprotein-cholesterol

We performed 2 × 2 factorial MR analyses, splitting our participants into four groups based on the scores of antihypertensive drugs and lipid-lowering drugs, as depicted in Fig. 1. Thus, individuals were categorized into: both scores ≥ median (reference), lipid-lowering drug score < median (representing statin or PCSK9 inhibitors use), antihypertensive drug score < median (representing BBs or CCBs medication), and both scores < median (proxy for combined medications). Then we investigated the associations with the outcomes between these groups in logistic regression models with adjustment for age, sex, assessment center, SNP array, and top ten genetic principal components. The mean differences of LDL-C and SBP between each two groups were directly calculated and compared to estimate the effect of antihypertensive drugs and lipid-lowering drugs separately or jointly for therapy. To avoid biased estimates attributed to arbitrary dichotomization and to maximize power, we conducted the tests of the additive interaction and multiplicative interaction using the two scores as quantitative traits. The additive interactions were evaluated via calculating relative excess risk due to interaction (RERI) and 95% CI for each combination using bootstrap. RERI or 95%CI covering 0 indicates independent and additive effect (i.e., no interaction); RERI > 0 implies positive interaction or synergism, and RERI < 0 represents negative interaction or antagonism. Their multiplicative interactions were evaluated using interaction *P* value for the product term.

All analyses were performed using R (version 3.5.1; R Project for Statistical Computing). A two-tailed *P* value less than 0.05 was considered statistically significant.

### Sensitivity analyses

In additional analyses, we excluded individuals with third degree relationships, defined by a kinship coefficient of 0.0442. And to obtain more clinically relatable results, 2 × 2 factorial MR analyses were performed using genetic drug scores below 30th percentile (indicating medication use) and above 70th percentile (indicating non-medication) as proxies for each medication. Additionally, we used cis-expression quantitative trait loci (cis-eQTL) as the genetic proxies of these drugs, based on previous studies [30–32]. Accordingly, we excluded SNPs with *r*^2^ > 0.1 and conducted factorial MR. Finally, we conducted analyses by excluding participants who used lipid-lowering or antihypertensive drugs at baseline.

## Results

### Participant characteristic

Additional file 1: Table S3 shows the characteristic of the participants, with 423,821 participants in the UK Biobank. The mean age of the participants was 56.79 years, and 194,883 (46.0%) of them were males. The mean LDL-C and SBP values were 146.21 mg/dL and 141.03 mmHg, respectively. There were totally 59,497 CVD prevalent and incident events, including 45,306 CAD events.

### Instrument variables validation

For lipid-lowering drugs, we identified 5 and 11 genetic variants in the *HMGCR* and *PCSK9* genes as proxies for statin and PCSK9 inhibitors, respectively. For antihypertensive drugs, we selected 6 and 24 genetic variants as proxies for BBs and CCBs, respectively. The detailed information of genetic variants was elaborated in Additional file 1: Table S1. As expected, the genetic scores of lipid-lowering (statin and PCSK9 inhibitors) and antihypertensive drugs (BBs and CCBs) were significantly associated with LDL-C and SBP levels (Additional file 1: Table S4), explaining 0.30%, 0.65%, 0.05%, and 0.11% of the variance, corresponding to the *F* statistics of 258, 253, 37, and 20, which indicated weak instrument variables were not likely to influence our results (Additional file 1: Table S5). For the independence assumption, although there was significant association of statin score with BMI, we confirmed that the allocation to the 2 × 2 groups was indeed random by comparing the characteristics between groups, indicating that potential confounders were unlikely to have effects on the results (Table 1). Moreover, the results with additional adjustment for BMI were consistent and robust (Additional file 2: Fig. S9-S11). Regarding the exclusion restriction assumption, the intercept of the MR-Egger regression was not significant, suggested no evidence of pleiotropy. Additionally, we selected reliable and widely used genetic variants for the drugs, which improved our confidence in using these variants as genetic instruments since it is also an effective strategy for avoiding pleiotropy to use the genetic variants with well-defined biological function.

**Table 1:** Baseline characteristics by groups determined by the antihypertensive drug and lipid-lowering drug scores

	Both scores ≥ median	Antihypertensive drug score < median	Lipid-lowering drug score < median	Both scores < median
Statin and BBs				
*N*	112,918	101,147	110,944	98,812
Age, years	56.80 (7.95)	56.81 (7.98)	56.76 (7.98)	56.78 (7.99)
Male, *n* (%)	52,087 (46.1)	46,351 (45.8)	51,026 (46.0)	45,419 (46.0)
BMI, kg/m2	27.32 (4.75)	27.33 (4.75)	27.46 (4.79)	27.46 (4.79)
SBP, mm Hg	141.46 (20.67)	140.54 (20.65)	141.37 (20.59)	140.68 (20.60)
DBP, mmHg	84.51 (11.28)	83.95 (11.22)	84.44 (11.25)	83.98 (11.20)
LDL cholesterol, mg/dL	147.68 (33.87)	147.88 (33.66)	144.50 (33.31)	144.74 (33.18)
HDL cholesterol, mg/dL	56.52 (14.95)	56.20 (14.81)	56.25 (14.85)	55.95 (14.72)
Triglyceride, mg/dL	154.77 (90.54)	156.24 (91.49)	154.03 (89.54)	156.30 (91.55)
Smoking, *n* (%)	51,696 (45.9)	46,368 (46.0)	50,761 (45.9)	45,533 (46.3)
CRP, mg/dL	2.58 (4.36)	2.59 (4.35)	2.59 (4.38)	2.59 (4.37)
CAD, *n* (%)	12,285 (10.9)	10,723 (10.6)	11,901 (10.7)	10,397 (10.5)
CVD, *n* (%)	16,031 (14.2)	14,036 (13.9)	15,740 (14.2)	13,690 (13.9)
Statin and CCBs				
*N*	107,038	107,027	104,878	104,878
Age, years	56.80 (7.96)	56.81 (7.97)	56.77 (7.99)	56.77 (7.98)
Male, *n* (%)	49,252 (46.0)	49,186 (46.0)	48,249 (46.0)	48,196 (46.0)
BMI, kg/m2	27.31 (4.73)	27.35 (4.77)	27.45 (4.79)	27.47 (4.78)
SBP, mm Hg	141.61 (20.73)	140.44 (20.58)	141.60 (20.68)	140.49 (20.50)
DBP, mmHg	84.55 (11.31)	83.94 (11.19)	84.51 (11.24)	83.94 (11.21)
LDL cholesterol, mg/dL	147.57 (33.78)	147.98 (33.77)	144.40 (33.12)	144.82 (33.38)
HDL cholesterol, mg/dL	56.35 (14.84)	56.39 (14.93)	56.13 (14.77)	56.09 (14.81)
Triglyceride, mg/dL	154.95 (90.62)	155.97 (91.36)	154.41 (89.98)	155.79 (91.02)
Smoking, *n* (%)	48,992 (45.9)	49,072 (46.0)	48,052 (46.0)	48,242 (46.2)
CRP, mg/dL	2.58 (4.39)	2.59 (4.32)	2.57 (4.34)	2.61 (4.41)
CAD, *n* (%)	11,800 (11.0)	11,208 (10.5)	11,298 (10.8)	11,000 (10.5)
CVD, *n* (%)	15,292 (14.3)	14,775 (13.8)	14,909 (14.2)	14,521 (13.8)
PCSK9 inhibitor and BBs				
*N*	111,970	100,018	111,892	99,941
Age, years	56.77 (7.96)	56.78 (7.99)	56.78 (7.97)	56.81 (7.98)
Male, *n* (%)	51,546 (46.0)	45,714 (45.7)	51,567 (46.1)	46,056 (46.1)
BMI, kg/m2	27.38 (4.77)	27.41 (4.78)	27.41 (4.76)	27.39 (4.77)
SBP, mm Hg	141.42 (20.66)	140.64 (20.59)	141.41 (20.60)	140.57 (20.66)
DBP, mmHg	84.46 (11.25)	84.01 (11.22)	84.49 (11.28)	83.92 (11.20)
LDL cholesterol, mg/dL	147.61 (33.83)	148.03 (33.73)	144.61 (33.37)	144.62 (33.11)
HDL cholesterol, mg/dL	56.43 (14.89)	56.13 (14.76)	56.35 (14.91)	56.02 (14.77)
Triglyceride, mg/dL	154.02 (89.82)	156.43 (91.60)	154.79 (90.28)	156.10 (91.44)
Smoking, *n* (%)	51,273 (46.0)	45,988 (46.1)	51,184 (45.9)	45,913 (46.1)
CRP, mg/dL	2.58 (4.39)	2.58 (4.32)	2.59 (4.34)	2.61 (4.41)
CAD, *n* (%)	12,323 (11.0)	10,655 (10.7)	11,863 (10.6)	10,465 (10.5)
CVD, *n* (%)	16,111 (14.4)	13,994 (14.0)	15,660 (14.0)	13,732 (13.7)
PCSK9 inhibitor and CCBs				
*N*	105,995	105,993	105,920	105,913
Age, years	56.79 (7.98)	56.77 (7.97)	56.79 (7.98)	56.81 (7.97)
Male, *n* (%)	48,611 (45.9)	48,649 (45.9)	48,894 (46.2)	48,729 (46.0)
BMI, kg/m2	27.39 (4.78)	27.39 (4.77)	27.37 (4.75)	27.43 (4.78)
SBP, mm Hg	141.65 (20.72)	140.46 (20.52)	141.56 (20.69)	140.47 (20.56)
DBP, mmHg	84.56 (11.28)	83.93 (11.19)	84.50 (11.27)	83.95 (11.22)
LDL cholesterol, mg/dL	147.59 (33.79)	148.02 (33.77)	144.42 (33.12)	144.81 (33.37)
HDL cholesterol, mg/dL	56.27 (14.77)	56.30 (14.88)	56.22 (14.84)	56.17 (14.86)
Triglyceride, mg/dL	154.58 (90.30)	155.74 (91.04)	154.79 (90.28)	156.02 (91.37)
Smoking, *n* (%)	48,454 (45.9)	48,807 (46.2)	48,577 (46.0)	48,520 (46.0)
CRP, mg/dL	2.56 (4.34)	2.59 (4.38)	2.59 (4.40)	2.61 (4.35)
CAD, *n* (%)	11,761 (11.1)	11,217 (10.6)	11,334 (10.7)	10,994 (10.4)
CVD, *n* (%)	15,332 (14.5)	14,773 (13.9)	14,870 (14.0)	14,522 (13.7)

“Both scores ≥ median” represented the group of placebos, “Antihypertensive drug score < median” was the proxy for antihypertensive medication use, “Lipid-lowering drug score < median” was the proxy for lipid-lowering medication use, and “both scores < median” represented combined medications. Continuous variables are presented as mean (standard deviation) and categorical variables as *n* (%) unless otherwise stated

*BMI* body mass index, *SBP* systolic blood pressure, *DBP* diastolic blood pressure, *LDL* low-density lipoprotein, *HDL* high-density lipoprotein, *CRP* C-reactive protein, *CAD* coronary artery disease, *CVD* cardiovascular disease

### Factorial analyses

Table 1 shows the baseline characteristic of the four groups dichotomized by genetic risk scores. There were no significant differences in any non-lipid or non-blood pressure related baseline characteristics between the groups, which was consistent with random partitioning of participants into each group by the lipid-lowering and antihypertensive drug genetic scores, showing that the allocation was indeed random.

**Fig. 2:**
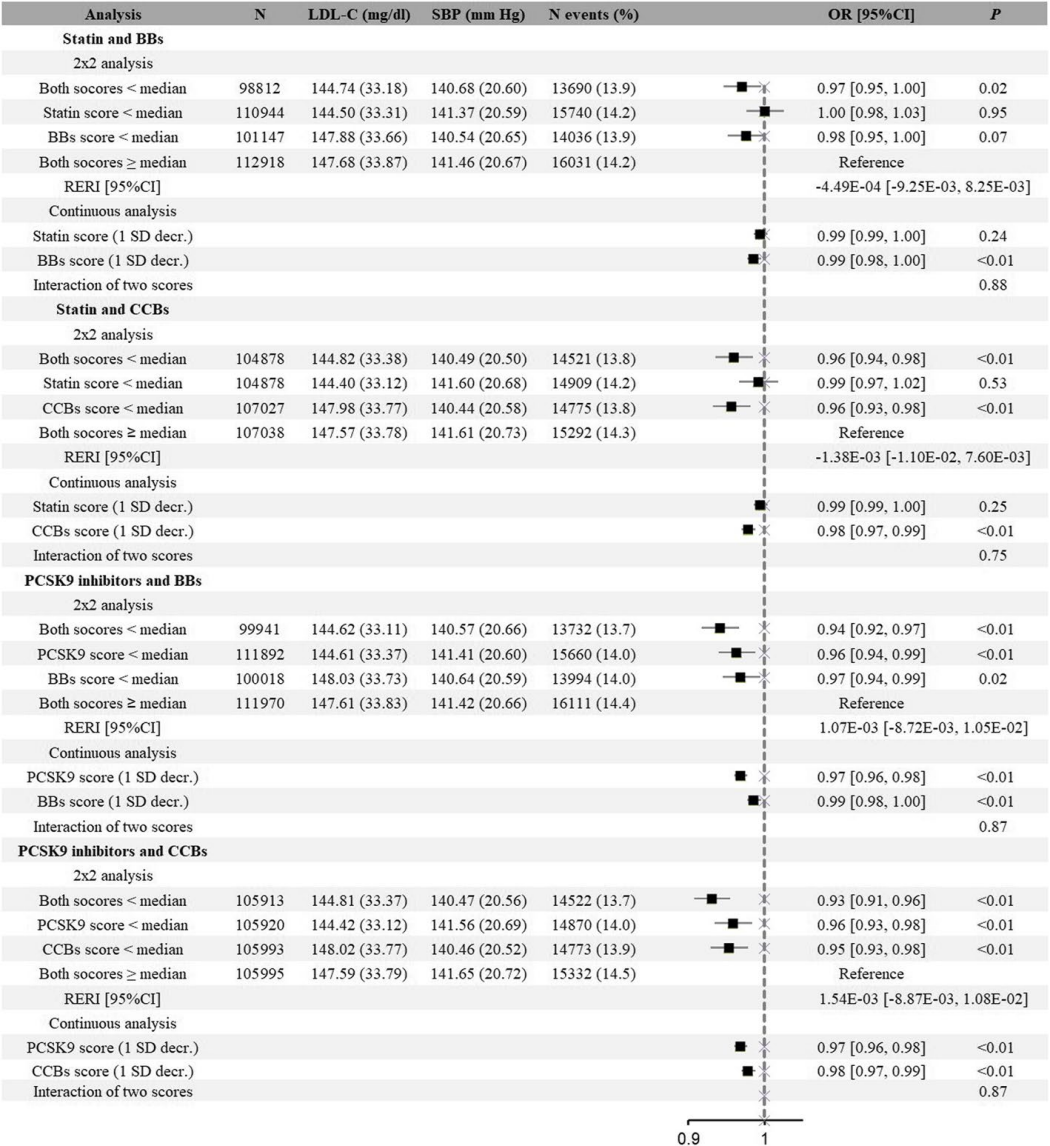
Associations of exposure to genetically proxied lipid-lowering drugs, genetically proxied antihypertensive drugs, or both with risk of cardiovascular disease. BBs beta-blockers, CCBs calcium channel blockers, RERI relative excess risk due to interaction. Notes: The 2 × 2 analysis divided participants into four groups according to the median genetic scores of lipid-lowering drugs and antihypertensive drugs: “Both scores ≥ median” represented the group of placebos, “statin/PCSK9 score < median” was the proxy for statin or PCSK9 inhibitors use; “BBs/CCBs score < median” was the proxy for BBs or CCBs medication; and “both scores < median” was the proxy for combined medications. Continuous analysis refers to the associations where the two genetic scores were included on a continuous scale (per SD decrease) as well as the interaction between the two scores

For CVD, we observed independent and additive effects in all combinations. In the 2 × 2 factorial MR analyses, either a lower genetic score of lipid-lowering drugs or antihypertensive drugs was associated with a lower risk of CVD, and scoring less than the median in both scores showed an approximately additive lower risk. When we performed analyses using the continuous genetic scores, there showed similar results as the dichotomized scores, and no interactions were observed. For instance, compared with the reference, 3.00 mg/dl lower LDL-C and a 4% lower risk of CVD were observed in individuals with a lower score of PCSK9 inhibitors (OR: 0.96; 95% CI: 0.94 to 0.99), and the individuals with a lower BBs score had 0.78 mmHg lower SBP and a 3% lower risk of CVD (OR: 0.97; 95% CI: 0.94 to 0.99); combined both had 2.99 mg/dl lower LDL-C, 0.85 mmHg lower SBP and was associated with a 6% lower risk of CVD (OR: 0.94; 95% CI: 0.92 to 0.97). This corresponded to a RERI index of 0 indicating an absolute lack of additive interaction (Fig. 2). In the continuous analyses, lower scores were also independently associated with a lower risk of CVD, with no evidence of multiplicative interaction between each 2 scores (*P* = 0.87 for interaction) (Fig. 2). Directionally consistent results for other drug combinations were similarly presented in Fig. 2. We found no significant interactions in the association of each two drugs with risk of CVD.

**Fig. 3:**
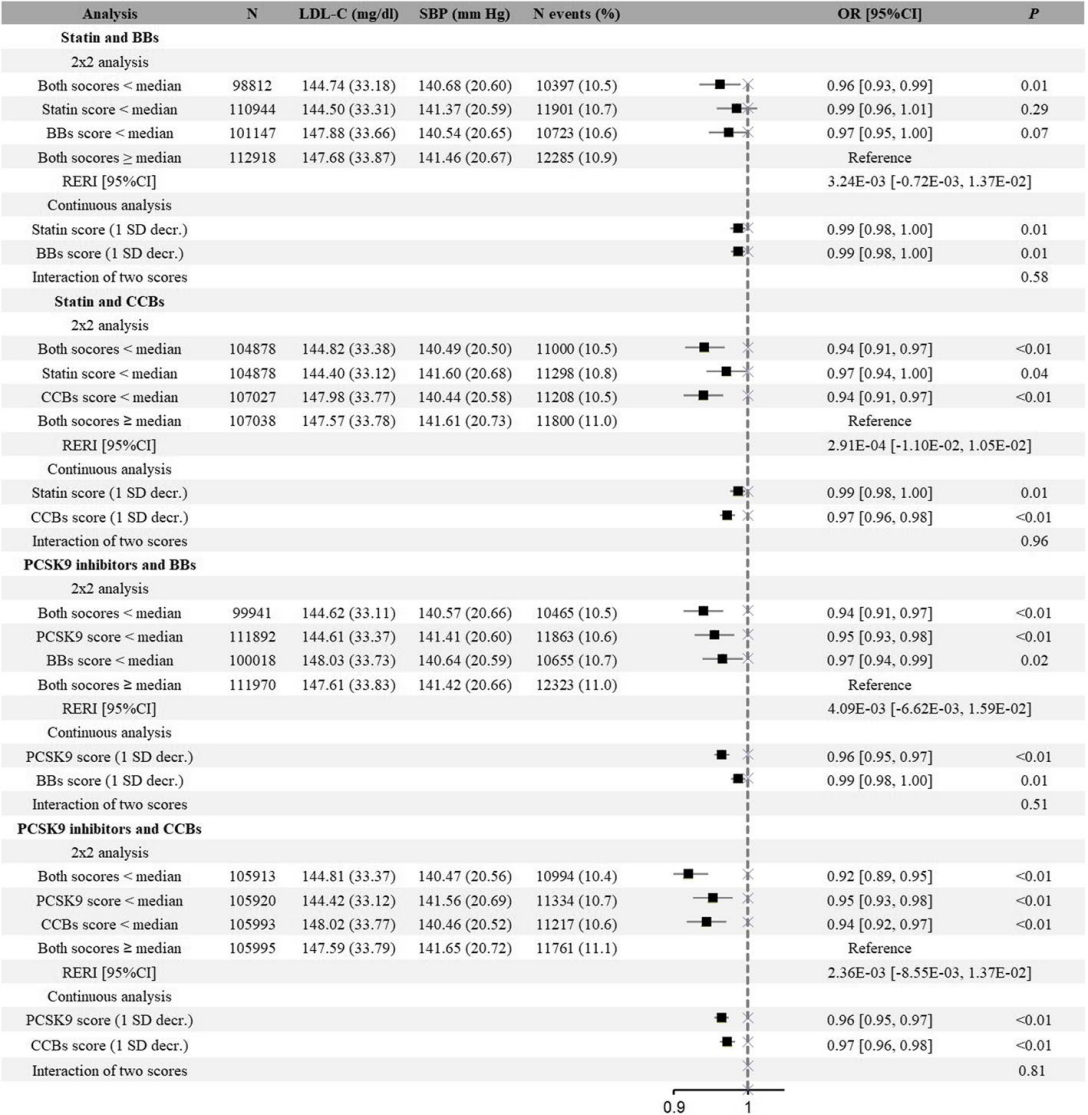
Associations of exposure to genetically proxied lipid-lowering drugs, genetically proxied antihypertensive drugs, or both with risk of coronary artery disease**.** BBs beta-blockers, CCBs calcium channel blockers, RERI relative excess risk due to interaction. Notes: The 2 × 2 analysis divided participants into four groups according to the median genetic scores of lipid-lowering drugs and antihypertensive drugs: “Both scores ≥ median” represented the group of placebos, “statin/PCSK9 score < median” was the proxy for statin or PCSK9 inhibitors use, “BBs/CCBs score < median” was the proxy for BBs or CCBs medication, and “both scores < median” was the proxy for combined medications. Continuous analysis refers to the associations where the two genetic scores were included on a continuous scale (per SD decrease) as well as the interaction between the two scores

**Fig. 4:**
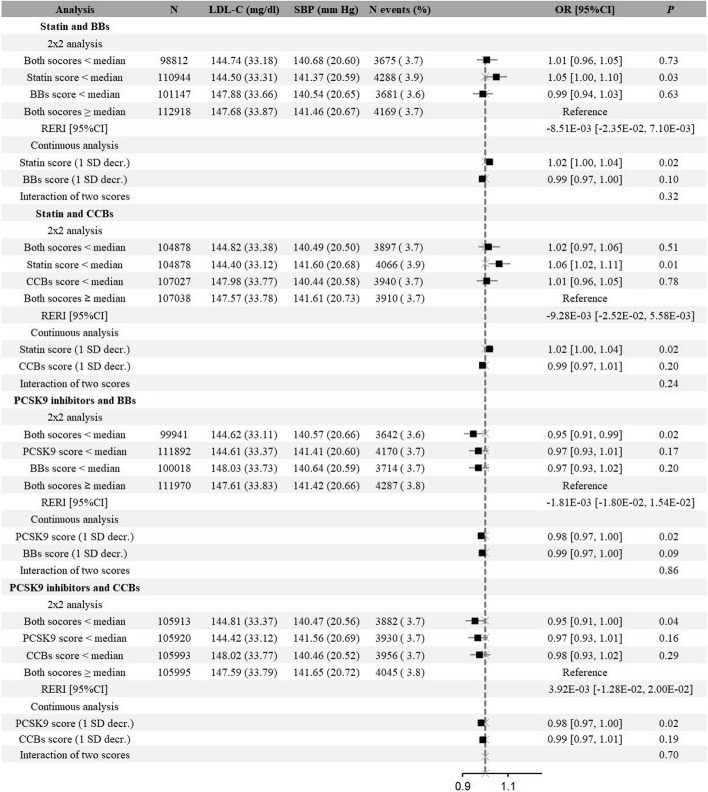
Associations of exposure to genetically proxied lipid-lowering drugs, genetically proxied antihypertensive drugs, or both with risk of stroke. BBs beta-blockers, CCBs calcium channel blockers, RERI relative excess risk due to interaction. Notes: The 2 × 2 analysis divided participants into four groups according to the median genetic scores of lipid-lowering drugs and antihypertensive drugs: “Both scores ≥ median” represented the group of placebos, “statin/PCSK9 score < median” was the proxy for statin or PCSK9 inhibitors use, “BBs/CCBs score < median” was the proxy for BBs or CCBs medication, and “both scores < median” was the proxy for combined medications. Continuous analysis refers to the associations where the two genetic scores were included on a continuous scale (per SD decrease) as well as the interaction between the two scores

As for other clinical and safety outcomes (including CAD, stroke, IS, HS, HF, DM, CKD, dementia, and PAD), similar additive effects were observed (Figs. 3, 4 and Additional file 2: Fig. S2-S8). Take CAD for example, genetically proxied medication of PCSK9 inhibitors was associated with decreased risk, and this association was not modified by BBs compared with the reference group (PCSK9 inhibitors: OR, 0.95; 95%CI, 0.93–0.98; BBs: OR, 0.97; 95%CI, 0.94–0.99; combined: OR, 0.94; 95%CI, 0.91–0.97; *P* = 0.51 for interaction). However, it is worth mentioning that when lipid-lowering and antihypertensive drugs were combined, there showed potential synergistic effect on DM, although it is not significant. A lower statin genetic score was associated with 4% higher risk for DM (OR: 1.04; 95% CI: 1.00 to 1.08), and a lower BBs score was associated with 5% higher risk for DM (OR: 1.05; 95% CI: 1.01 to 1.10); whereas combined both was associated with 12% higher risk for DM (OR: 1.12; 95% CI: 1.08 to 1.17) (Additional file 2: Fig. S5).

### Sensitivity analyses

We observed consistent results when the individuals with third degree relationship were further excluded (Additional file 2: Fig. S12-S14). For the analyses grouping by 30th and 70th percentile, the results were consistent with the main analyses and the effect sizes were larger (Additional file 2: Fig. S15-S17). Regarding the factorial MR using cis-eQTL as the genetic proxies, substantially consistent results were observed. The interaction was significant when proxies for PCSK9 inhibitors and BBs were combined for CVD (Additional file 2: Fig. S18-S20). The results of excluding participants taking antihypertensive and lipid-lowering drugs were similar to the main analyses (Additional file 2: Fig. S21-S23).

## Discussion

We conducted factorial MR analyses to evaluate the effects of naturally random allocation to genetically lower blood lipids (mediated by targets of statin or PCSK9 inhibitors), lower blood pressure (mediated by targets of BBs or CCBs), or combined both on the risk of CVD and other clinical outcomes based on the UK Biobank. Our results suggest that the combination of lipid-lowering and antihypertensive drugs will result in an independent and additive effect on the risk of CVD and other clinical outcomes. These findings were with implications for the polypill development and clinical trials which need compare the proportion of drug compounds of joint prescription. Our study has potential to inform the therapeutic strategies towards the primary and secondary prevention of CVD.

For CVD, prior studies have illustrated the effectiveness of lipid-lowering drugs and antihypertensive drugs on reducing risk of CVD [33–35], but few studies explored their joint effects. In this study, we not only evaluated the effect of antihypertensive drugs, lipid-lowering drugs, and combined medications but assessed the interactions between the two drugs on CVD and clinically related outcomes. As expected, we found that either lower LDL-C mediated by targets of lipid-lowering drugs (statins and PCSK9 inhibitors) or lower SBP mediated by targets of antihypertensive drugs (BBs and CCBs) was causally associated with lower risk of CVD, which was consistent with previous RCT [5, 36]. And our results suggested no interactions of antihypertensive drugs and lipid-lowering drugs on risk of CVD, other clinical and safety outcomes, which apparently exhibited independent and additive effect. This suggests that although the targets of each drug affect CVD and other disease risks, they might act through distinct pathways and therefore present additive effects, which need further studies to explore the underlying mechanism. In both our study of medications and the study by Ference et al. concerning SBP and LDL-C levels [14], no interactions were found, indicating consistent findings. Although previously a few experiments indicated there were potential pharmacological synergism between statin and antihypertensive drugs [10, 37], the findings in other studies were inconsistent [13, 38]. When we conducted analyses considering the genetic scores as continuous variables, the results still indicated no interactions between the combinations of any two drugs and were also consistent with our 2×2 factorial analyses. This indicated that it may be better to focus more on the therapy of hypertension and dyslipidemia with the corresponding single drugs for the population benefit comprehensively [11]. The sensitivity analyses using eQTL related to gene expression as genetic proxies indicated significant interactions between PCSK9 inhibitors and BBs on CVD, which need further studies to validate. For the drug development of polypills which could reduce cardiovascular risk and improve adherence [39, 40], our findings can also inform the proportion of drug composition and analysis of drug efficacy. In addition, these findings could provide genetic evidence for the subsequent 2 × 2 RCT.

For other clinical and safety outcomes, there also exhibited additive magnitude between lipid-lowering and antihypertensive drugs. Therefore, when joint prescription of the two drugs, the additive risk of adverse events should be usually concerned. We observed a potential synergistic effect on DM risk when statin and BBs were genetically combined, indicating that more attention should be paid to the status of patients’ blood glucose level during the clinical application of these two drugs.

There were several strengths in our study. First, factorial MR could avoid residual confounding and reverse causation and simulate the effects of factorial RCT without clinical interventions. Second, most of the 2 × 2 factorial RCT combining the two drugs focused on outcomes of functional and biochemical indicators such as endothelial function which could be observed in a comparatively shorter time [8, 41, 42], while we focused on the outcomes of CVD and clinically relevant diseases and assessed interactions between the two drugs by 2 × 2 factorial MR.

However, there were also several limitations in this study. First, the statistical power of some outcomes was relatively low because of the small number of individuals with disease, which may lead to some false-negative results (e.g., antihypertensive drugs on HS risk, which has a smaller number of events, requiring higher power). Second, the strength of evidence in the factorial MR is not as strong as in the RCT, and the effect of factorial MR may differ from the short-term and intensive pharmacological modulation in RCT or clinical practice because factorial MR utilize genetic proxies of drug targets as instrument variables rather than focusing on the participants actually taking these drugs in real-life. Third, our study included only Europeans which might limit extrapolation to other ethnic populations. In addition, the remaining few lipid-lowering and antihypertensive drugs could not be explored in our study. Therefore, larger-scale studies including clinical trials of drug interventions in multiethnic populations are still required to explore interactions between the two drugs and the best drug combinations for populations to inform the clinical medication and drug development of polypills.

## Conclusions

We conducted 2 × 2 factorial MR analyses and found that combined medication of lipid-lowering and antihypertensive drugs had additive effects on risk of CVD and other clinical diseases. Our findings provide a deeper understanding of clinical practice in those with hypertension and dyslipidemia. Further trials are warranted to explore combined therapeutics with lipid-lowering and antihypertensive drugs on cardiovascular benefits.

### Supplementary Information

Additional file 1: Table S1. Genetic variants included in the genetic risk scores. Table S2. Ascertainment of cardiovascular diseases and other clinical outcomes in the UK Biobank. Table S3. Summary Demographic and Outcome Information. Table S4. Associations of the genetic scores for lipid-lowering drugs and antihypertensive drugs with measured LDL-C levels, SBP levels, CVD, and other clinical outcomes. Table S5. Strength of genetic instruments for the lipid-lowering and antihypertensive drugs. Table S6. Genetic variants included in the genetic scores of expression quantitative trait locus (eQTL).

Additional file 2: Fig. S1. Participant flow chart of the study in the UK Biobank. Fig. S2. Associations of exposure to genetically proxied lipid-lowering drugs, genetically proxied antihypertensive drugs, or both with risk of ischemic stroke. Fig. S3. Associations of exposure to genetically proxied lipid-lowering drugs, genetically proxied antihypertensive drugs, or both with risk of hemorrhagic stroke. Fig. S4. Associations of exposure to genetically proxied lipid-lowering drugs, genetically proxied antihypertensive drugs, or both with risk of heart failure. Fig. S5. Associations of exposure to genetically proxied lipid-lowering drugs, genetically proxied antihypertensive drugs, or both with risk of diabetes mellitus. Fig. S6. Associations of exposure to genetically proxied lipid-lowering drugs, genetically proxied antihypertensive drugs, or both with risk of chronic kidney disease. Fig. S7. Associations of exposure to genetically proxied lipid-lowering drugs, genetically proxied antihypertensive drugs, or both with risk of dementia. Fig. S8. Associations of exposure to genetically proxied lipid-lowering drugs, genetically proxied antihypertensive drugs, or both with risk of peripheral artery diseases. Fig. S9. Associations of exposure to genetically proxied lipid-lowering drugs, genetically proxied antihypertensive drugs, or both with risk of cardiovascular diseases, with additional adjustment for body mass index. Fig. S10. Associations of exposure to genetically proxied lipid-lowering drugs, genetically proxied antihypertensive drugs, or both with risk of coronary artery diseases, with additional adjustment for body mass index. Fig. S11. Associations of exposure to genetically proxied lipid-lowering drugs, genetically proxied antihypertensive drugs, or both with risk of stroke, with additional adjustment for body mass index. Fig. S12. Associations of exposure to genetically proxied lipid-lowering drugs, genetically proxied antihypertensive drugs, or both with risk of cardiovascular disease, as defined by a kinship coefficient of 0.0442. Fig. S13. Associations of exposure to genetically proxied lipid-lowering drugs, genetically proxied antihypertensive drugs, or both with risk of coronary artery disease, as defined by a kinship coefficient of 0.0442. Fig. S14. Associations of exposure to genetically proxied lipid-lowering drugs, genetically proxied antihypertensive drugs, or both with risk of stroke, as defined by a kinship coefficient of 0.0442. Fig. S15. Associations of exposure to genetically proxied lipid-lowering drugs, genetically proxied antihypertensive drugs, or both with risk of cardiovascular disease, using 30th percentiles and 70th percentile for grouping. Fig. S16. Associations of exposure to genetically proxied lipid-lowering drugs, genetically proxied antihypertensive drugs, or both with risk of coronary artery disease, using 30th percentiles and 70th percentile for grouping. Fig. S17. Associations of exposure to genetically proxied lipid-lowering drugs, genetically proxied antihypertensive drugs, or both with risk of stroke, using 30th percentiles and 70th percentile for grouping. Fig. S18. Associations of exposure to genetically proxied lipid-lowering drugs, genetically proxied antihypertensive drugs, or both with risk of cardiovascular diseases, with eQTL as the genetic proxies for these four drugs. Fig. S19. Associations of exposure to genetically proxied lipid-lowering drugs, genetically proxied antihypertensive drugs, or both with risk of coronary artery disease, with eQTL as the genetic proxies for these four drugs. Fig. S20. Associations of exposure to genetically proxied lipid-lowering drugs, genetically proxied antihypertensive drugs, or both with risk of stroke, with eQTL as the genetic proxies for these four drugs. Fig. S21. Associations of exposure to genetically proxied lipid-lowering drugs, genetically proxied antihypertensive drugs, or both with risk of cardiovascular disease, excluding participants who used lipid-lowering or antihypertensive drugs at baseline. Fig. S22. Associations of exposure to genetically proxied lipid-lowering drugs, genetically proxied antihypertensive drugs, or both with risk of coronary artery disease, excluding participants who used lipid-lowering or antihypertensive drugs at baseline. Fig. S23. Associations of exposure to genetically proxied lipid-lowering drugs, genetically proxied antihypertensive drugs, or both with risk of stroke, excluding participants who used lipid-lowering or antihypertensive drugs at baseline.

## Data Availability

The UK Biobank data are available on request from the UK Biobank website (www.ukbiobank.ac.uk/). All used in the present study are described in the “Methods” section and presented in Additional file 1 and  Additional file 2.

## Abbreviations

ACEI: Angiotensin-converting enzyme inhibitors
BBs: β-Blockers
BMI: Body mass index
CCBs: Calcium channel blockers
CKD: Chronic kidney disease
CVD: Cardiovascular disease
DM: Diabetes mellitus
eQTL: Expression quantitative trait loci
GWAS: Genome-wide association study
HF: Heart failure
HS: Hemorrhagic stroke
ICD: International Classification of Disease
IS: Ischemic stroke
LDL-C: Low-density lipoprotein cholesterol
MR: Mendelian randomization
OR: Odds ratio
PAD: Peripheral artery diseases
PCSK9: Proprotein convertase subtilisin/kexin type 9
RCT: Randomized controlled trials
RERI: Relative excess risk due to interaction
SBP: Systolic blood pressure
SNP: Single-nucleotide polymorphism
