# Supplementary material for: Association of genetic variants related to combined lipid-lowering and antihypertensive therapies with risk of cardiovascular disease: 2 × 2 factorial Mendelian randomization analyses
Source: BMC Med. 2024 May 20;22:201. doi: 10.1186/s12916-024-03407-x (PMC11103938; doi:10.1186/s12916-024-03407-x)
Supplement: Supplementary file 1 — Additional file 1: Table S1. Genetic variants included in the genetic risk scores. Table S2. Ascertainment of cardiovascular diseases and other clinical outcomes in the UK Biobank. Table S3. Summary Demographic and Outcome Information. Table S4. Associations of the genetic scores for lipid-lowering drugs and antihypertensive drugs with measured LDL-C levels, SBP levels, CVD, and other clinical outcomes. Table S5. Strength of genetic instruments for the lipid-lowering and antihypertensive drugs. Table S6. Genetic variants included in the genetic scores of expression quantitative trait locus (eQTL). [file 12916_2024_3407_MOESM1_ESM.docx]

# Additional file1

**Table S1. Genetic variants included in the genetic risk scores.**

| Drug | SNP | CHR | POS | BETA | SE | *P*-value | EAF | EA | OA |
| --- | --- | --- | --- | --- | --- | --- | --- | --- | --- |
| Statins | rs10515198 | 5 | 74641560 | 0.060 | 0.006 | 5.99E-22 | 0.103 | A | G |
| Statins | rs12173076 | 5 | 74697050 | 0.065 | 0.006 | 2.33E-27 | 0.123 | G | T |
| Statins | rs12916 | 5 | 74656539 | 0.073 | 0.004 | 7.79E-78 | 0.431 | C | T |
| Statins | rs3857388 | 5 | 74620377 | 0.042 | 0.006 | 2.20E-11 | 0.128 | C | T |
| Statins | rs7711235 | 5 | 74540397 | 0.038 | 0.006 | 5.00E-10 | 0.268 | G | A |
| PCSK9 inhibitors | rs10493176 | 1 | 55538552 | -0.078 | 0.010 | 2.54E-14 | 0.115 | G | T |
| PCSK9 inhibitors | rs11206510 | 1 | 55496039 | -0.083 | 0.005 | 2.38E-53 | 0.154 | C | T |
| PCSK9 inhibitors | rs11206514 | 1 | 55516004 | 0.051 | 0.004 | 9.95E-33 | 0.611 | A | C |
| PCSK9 inhibitors | rs11583974 | 1 | 55551718 | 0.065 | 0.012 | 3.95E-09 | 0.030 | A | G |
| PCSK9 inhibitors | rs11591147 | 1 | 55505647 | -0.497 | 0.018 | 8.57E-143 | 0.017 | T | G |
| PCSK9 inhibitors | rs12067569 | 1 | 55528629 | 0.089 | 0.010 | 1.97E-17 | 0.034 | A | G |
| PCSK9 inhibitors | rs2479394 | 1 | 55486064 | -0.039 | 0.004 | 1.58E-19 | 0.715 | A | G |
| PCSK9 inhibitors | rs2479409 | 1 | 55504650 | -0.064 | 0.004 | 2.51E-50 | 0.668 | A | G |
| PCSK9 inhibitors | rs2495477 | 1 | 55518467 | -0.064 | 0.005 | 7.28E-30 | 0.400 | G | A |
| PCSK9 inhibitors | rs572512 | 1 | 55517344 | 0.048 | 0.005 | 5.31E-26 | 0.346 | T | C |
| PCSK9 inhibitors | rs585131 | 1 | 55524116 | 0.064 | 0.005 | 2.70E-35 | 0.815 | T | C |
| BBs | rs11196549 | 10 | 115707298 | 0.688 | 0.078 | 1.58E-18 | 0.043 | A | G |
| BBs | rs460718 | 10 | 115721364 | -0.276 | 0.032 | 1.36E-17 | 0.327 | A | G |
| BBs | rs11196597 | 10 | 115788094 | 0.286 | 0.046 | 4.23E-10 | 0.133 | A | G |
| BBs | rs17875473 | 10 | 115800294 | 0.328 | 0.055 | 2.66E-09 | 0.087 | T | C |
| BBs | rs1801253 | 10 | 115805056 | 0.463 | 0.034 | 2.84E-41 | 0.734 | C | G |
| BBs | rs4359161 | 10 | 115826508 | -0.266 | 0.039 | 9.46E-12 | 0.181 | A | G |
| CCBs | rs3821843 | 3 | 53558012 | 0.337 | 0.034 | 6.56E-24 | 0.681 | A | G |
| CCBs | rs114987861 | 3 | 53605712 | 0.529 | 0.096 | 3.36E-08 | 0.028 | A | G |
| CCBs | rs113210396 | 3 | 53612327 | -0.434 | 0.077 | 1.76E-08 | 0.045 | T | G |
| CCBs | rs7340705 | 3 | 53734443 | -0.243 | 0.032 | 4.87E-14 | 0.673 | T | C |
| CCBs | rs2488136 | 10 | 18334521 | 0.226 | 0.033 | 1.22E-11 | 0.288 | A | G |
| CCBs | rs1888693 | 10 | 18440444 | 0.386 | 0.032 | 4.69E-34 | 0.345 | A | G |
| CCBs | rs16916914 | 10 | 18457722 | -0.564 | 0.081 | 2.72E-12 | 0.963 | T | C |
| CCBs | rs7076319 | 10 | 18459450 | -0.321 | 0.034 | 5.07E-21 | 0.734 | A | G |
| CCBs | rs61278674 | 10 | 18481737 | -0.330 | 0.054 | 1.03E-09 | 0.906 | A | G |
| CCBs | rs1779209 | 10 | 18514561 | 0.274 | 0.034 | 4.23E-16 | 0.288 | T | C |
| CCBs | rs10828399 | 10 | 18553968 | -0.195 | 0.030 | 1.10E-10 | 0.522 | A | G |
| CCBs | rs10828452 | 10 | 18592450 | 0.305 | 0.039 | 4.20E-15 | 0.793 | A | T |
| CCBs | rs10828542 | 10 | 18627285 | 0.182 | 0.031 | 5.18E-09 | 0.614 | A | G |
| CCBs | rs12780039 | 10 | 18678987 | 0.285 | 0.047 | 1.26E-09 | 0.121 | C | G |
| CCBs | rs112133583 | 10 | 18695681 | -0.555 | 0.097 | 1.18E-08 | 0.030 | T | C |
| CCBs | rs11014170 | 10 | 18710991 | -0.670 | 0.115 | 5.61E-09 | 0.021 | A | G |
| CCBs | rs7923191 | 10 | 18727901 | -0.369 | 0.038 | 1.10E-22 | 0.792 | A | G |
| CCBs | rs12258967 | 10 | 18727959 | 0.633 | 0.034 | 1.08E-78 | 0.705 | C | G |
| CCBs | rs72786098 | 10 | 18729855 | -0.503 | 0.088 | 1.18E-08 | 0.032 | A | G |
| CCBs | rs1998822 | 10 | 18755664 | -0.196 | 0.034 | 1.15E-08 | 0.723 | A | G |
| CCBs | rs4748474 | 10 | 18790727 | 0.195 | 0.030 | 1.61E-10 | 0.521 | A | G |
| CCBs | rs150857355 | 12 | 49209340 | 0.941 | 0.112 | 5.20E-17 | 0.022 | C | G |
| CCBs | rs2239046 | 12 | 2434419 | 0.208 | 0.032 | 9.58E-11 | 0.682 | A | G |
| CCBs | rs714277 | 12 | 2514270 | 0.199 | 0.033 | 2.38E-09 | 0.283 | T | C |

SNP, single-nucleotide polymorphism; CHR, chromosome; POS, position; SE, standard error; EAF, effect allele frequency; EA, effect allele; OA, other allele;

BBs, beta-blockers; CCBs, calcium channel blockers.

**Table S2**. **Ascertainment of cardiovascular diseases and other clinical outcomes in the UK Biobank.**

| Outcomes | Source | Code |
| --- | --- | --- |
| CAD | HES (ICD10) | I20, I21, I22, I23, I24, I25 |
|  | HES (ICD9) | 410, 411, 412, 413, 414, 415.1, 428, 429.2, 429.5, 429.6, 429.7 |
|  | HES (OPCs) | K40, K41, K42, K43, K44, K45, K46, K47.1, K49, K50, K59, K75, X50 |
|  | Baseline (non-cancer illness code, self-reported) | 1074, 1075 |
|  | Baseline (operation) | 1095, 1070 |
|  | Cause of death (ICD10 primary + secondary) | I20, I21, I22, I23, I24, I25 |
| PAD | HES (ICD10) | I73.1, I73.8, I73.9 |
|  | HES (ICD9) | 443.1, 443.8, 443.9 |
|  | HES (OPCs) | L50, L51, L52, L53, L54, L58, L59, L60, L62, L63, L65 |
|  | Baseline (non-cancer illness code, self-reported) | 1067, 1087, 1492, 1591 |
|  | Baseline (operation) | 1071, 1102, 1103, 1104, 1107, 1108, 1515 |
|  | Cause of death (ICD10 primary + secondary) | I73.1, I73.8, I73.9 |
| Stroke | HES (ICD10) | G46.3, G46.4, G46.5, G46.6, G46.7, I60, I61, I62.0, I62.1, I62.9, I63, I64, I69.0, I69.1, I69.2, I69.3, I69.4, I69.8 |
|  | HES (ICD9) | 430, 431, 432.0, 432.1, 432.9, 433, 434, 436, 437.0, 437.9 |
|  | HES (OPCs) | U54.3 |
|  | Baseline: Vascular problem code | 3 |
|  | Baseline: Non cancer illness code | 1081, 1082, 1083, 1086, 1491, 1583 |
|  | Cause of death ICD10 (Primary + Secondary) | G46.3, G46.4, G46.5, G46.6, G46.7, I60, I61, I62.0, I62.1, I62.9, I63, I64, I69.0, I69.1, I69.2, I69.3, I69.4, I69.8 |
| Hemorrhagic stroke | HES (ICD10) | I60, I61, I62 |
|  | HES (ICD9) | 430, 431, 432 |
|  | Baseline (non-cancer illness code) | 1086, 1083, 1491 |
|  | Cause of death (ICD10 primary + secondary) | I60, I61, I62 |
| Ischemic stroke | HES (ICD10) | I63, I64 |
|  | HES (ICD9) | 434, 436 |
|  | Baseline (non-cancer illness code, self-reported) | 1583 |
|  | Cause of death (ICD10 primary + secondary) | I63, I64 |
| Diabetes mellitus | HES (ICD10) | E11, E14 |
|  | HES (ICD9) | 250.00, 250.02, 250.10, 250.12, 250.20, 250.22, 250.30, 250.32, 250.40, 250.42, 250.50, 250.52, 250.60,250.62, 250.70, 250.72, 250.80, 250.82, 250.90, 250.92 |
|  | Baseline (non-cancer illness code, self-reported) | 1220, 1223 |
|  | Cause of death (ICD10 primary + secondary) | E11, E14 |
| Dementia | HES (ICD10) | F00, F01, F02, F03, G30, A810, G310, G311, G318, F051, F106 |
|  | HES (ICD9) | 331.0, 290.4, 331.1, 290.2, 290.3, 291.2, 294.1, 331.2, 331.5 |
|  | Baseline (non-cancer illness code) | 1263 |
|  | Cause of death (ICD10 primary + secondary) | F00, F01, F02, F03, G30, A810, G310, G311, G318, F051, F106 |
| Chronic kidney disease | HES (ICD10) | I12.0, I13.1, I13.2, N18 |
|  | HES (ICD-9) | 585 |
|  | HES (OPCs) | M01 |
|  | Cause of death (ICD10 primary + secondary) | I12.0, I13.1, I13.2, N18 |
| Heart failure | HES (ICD-10) | I11.0, I13.0, I13.2, I25.5, I42.0, I42.1, I42.5, I42.8, I42.9, I50 |
|  | HES (ICD-9) | 4254, 4280, 4281, 4289 |
|  | Cause of death (ICD10 primary + secondary) | I11.0, I13.0, I13.2, I25.5, I42.0, I42.1, I42.5, I42.8, I42.9, I50 |

CAD, Coronary artery diseases; PAD, peripheral artery diseases; OPCS, Office of Population Censuses and Surveys Classification of Surgical Operations and Procedures Classification of Interventions and Procedures. (OPCS Classification of Interventions and Procedures version 4); HES, Hospital Episodes Statistics; ICD, International Classification of Disease.

Note: Those with CAD or PAD or stroke was considered as cardiovascular disease (CVD).

**Table S3. Summary Demographic and Outcome Information**

| Characteristic | UK Biobank (*N*=423,821) |
| --- | --- |
| Age at baseline, years | 56.79 (7.97) |
| Male, n (%) | 194,883 (46.0) |
| BMI, kg/m2 | 27.40 (4.77) |
| SBP, mmHg | 141.03 (20.63) |
| DBP, mmHg | 84.23 (11.24) |
| LDL Cholesterol, mg/dL | 146.21 (33.55) |
| HDL Cholesterol, mg/dL | 56.24 (14.84) |
| Triglyceride, mg/dL | 155.28 (90.75) |
| CAD, n (%) | 45,306 (10.7) |
| CVD, n (%) | 59,497 (14.0) |
| lipid-lowering medication use, n (%) | 73,151 (17.3) |
| antihypertensive medication use, n (%) | 87,345 (20.6) |

Continuous variables are presented as mean (standard deviation) and categorical variables as n (%) unless otherwise stated.

BMI, body mass index; SBP, systolic blood pressure; DBP, diastolic blood pressure; LDL, low-density lipoprotein; HDL, high-density lipoprotein; CAD, coronary artery disease; CVD, cardiovascular disease.

**Table S4. Associations of the genetic scores for lipid-lowering drugs and antihypertensive drugs with measured LDL-C levels, SBP levels, CVD, and other clinical outcomes.**

| Genetic risk score | trait | Beta | Standard Error | *P* value |
| --- | --- | --- | --- | --- |
| Statin | LDL-C | 0.047 | 0.001 | 3.85E-270 |
|  | CAD | 0.014 | 0.005 | 8.31E-03 |
|  | CVD | 0.005 | 0.005 | 2.45E-01 |
|  | IS | -0.012 | 0.012 | 3.17E-01 |
|  | HS | -0.053 | 0.017 | 2.30E-03 |
|  | HF | -0.018 | 0.014 | 1.88E-01 |
|  | DM | -0.022 | 0.007 | 2.37E-03 |
|  | CKD | 0.032 | 0.023 | 1.60E-01 |
|  | Dementia | -0.004 | 0.020 | 8.51E-01 |
|  | Stroke | -0.020 | 0.008 | 1.74E-02 |
|  | PAD | 0.009 | 0.014 | 5.47E-01 |
| PCSK9 inhibitor | LDL-C | 0.069 | 0.001 | 0 |
|  | CAD | 0.036 | 0.005 | 5.78E-12 |
|  | CVD | 0.031 | 0.005 | 1.01E-11 |
|  | IS | 0.023 | 0.012 | 4.37E-02 |
|  | HS | -0.012 | 0.017 | 4.88E-01 |
|  | HF | 0.006 | 0.014 | 6.55E-01 |
|  | DM | 0.005 | 0.007 | 4.48E-01 |
|  | CKD | 0.012 | 0.023 | 6.15E-01 |
|  | Dementia | -0.003 | 0.020 | 8.96E-01 |
|  | Stroke | 0.019 | 0.008 | 2.24E-02 |
|  | PAD | 0.030 | 0.014 | 3.58E-02 |
| BBs | SBP | 0.457 | 0.030 | 4.80E-51 |
|  | CAD | 0.013 | 0.005 | 9.71E-03 |
|  | CVD | 0.014 | 0.005 | 2.84E-03 |
|  | IS | 0.004 | 0.012 | 7.09E-01 |
|  | HS | 0.006 | 0.017 | 7.14E-01 |
|  | HF | 0.013 | 0.014 | 3.34E-01 |
|  | DM | -0.031 | 0.007 | 2.68E-05 |
|  | CKD | 0.003 | 0.023 | 9.09E-01 |
|  | Dementia | -0.008 | 0.020 | 6.93E-01 |
|  | Stroke | 0.014 | 0.008 | 9.32E-02 |
|  | PAD | 0.021 | 0.014 | 1.42E-01 |
| CCBs | SBP | 0.685 | 0.030 | 1.48E-113 |
|  | CAD | 0.028 | 0.005 | 4.90E-08 |
|  | CVD | 0.021 | 0.005 | 2.90E-06 |
|  | IS | 0.007 | 0.011 | 5.26E-01 |
|  | HS | 0.009 | 0.017 | 6.00E-01 |
|  | HF | 0.019 | 0.014 | 1.72E-01 |
|  | DM | -0.007 | 0.007 | 3.37E-01 |
|  | CKD | 0.010 | 0.023 | 6.77E-01 |
|  | Dementia | -0.024 | 0.020 | 2.39E-01 |
|  | Stroke | 0.011 | 0.008 | 1.92E-01 |
|  | PAD | -0.003 | 0.014 | 8.33E-01 |

CAD, coronary artery disease; CVD, cardiovascular disease; BBs: beta-blockers; CCBs, calcium channel blockers; SBP, systolic blood pressure; LDL-C, low-density lipoprotein-cholesterol; PAD, peripheral artery diseases; IS, ischemic stroke; HS, hemorrhagic stroke; HF, heart failure; DM, diabetes mellitus; CKD, chronic kidney disease.

**Table S5. Strength of genetic instruments for the lipid-lowering and antihypertensive drugs.**

| Drug | N | No. of SNPs to generate the gene scores | R^2^ (%) | F statistics |
| --- | --- | --- | --- | --- |
| Statins | 423,821 | 5 | 0.30 | 258 |
| PCSK9 inhibitors |  | 11 | 0.65 | 253 |
| BBs |  | 6 | 0.05 | 37 |
| CCBs |  | 24 | 0.11 | 20 |

PCSK9, proprotein convertase subtilisin–kexin type 9; BBs, β-blockers; CCBs, calcium channel blockers; N, sample size; SNP, single nucleotide polymorphisms; R^2^, variance explained by the genetic score; ^*^ F statistics was calculated by the formular F = (R^2^/K) / ((1 – R^2^) / (N-K-1)), where K = 1, and N = sample size.

**Table S6. Genetic variants included in the genetic scores of expression quantitative trait locus (eQTL).**

| Drug | SNP | BETA | SE | EA | OA | EAF |
| --- | --- | --- | --- | --- | --- | --- |
| BB | rs1383914 | -6.076E-03 | 2.453E-03 | C | T | 4.944E-01 |
| BB | rs184489 | -1.967E-02 | 9.273E-03 | C | T | 9.822E-01 |
| BB | rs1899494 | 1.054E-02 | 2.801E-03 | A | C | 2.605E-01 |
| BB | rs77185818 | -8.523E-03 | 3.794E-03 | A | G | 1.200E-01 |
| BB | rs1009890 | 5.230E-03 | 2.657E-03 | C | T | 6.855E-01 |
| BB | rs12654978 | -5.126E-03 | 2.594E-03 | T | C | 3.464E-01 |
| BB | rs13159674 | 8.472E-03 | 3.063E-03 | T | C | 2.021E-01 |
| BB | rs143589638 | -1.183E-02 | 5.550E-03 | G | A | 5.196E-02 |
| BB | rs17645325 | 1.444E-02 | 3.713E-03 | C | T | 1.251E-01 |
| BB | rs4815605 | -5.284E-03 | 2.482E-03 | A | G | 5.567E-01 |
| BB | rs55778060 | 1.075E-02 | 3.810E-03 | T | C | 1.250E-01 |
| BB | rs57698607 | 1.432E-02 | 5.309E-03 | T | C | 5.752E-02 |
| BB | rs735710 | -1.429E-02 | 4.855E-03 | C | T | 7.039E-02 |
| BB | rs11196589 | 5.045E-03 | 2.531E-03 | C | A | 4.039E-01 |
| BB | rs151591 | 1.245E-02 | 2.918E-03 | G | A | 7.674E-01 |
| BB | rs7076938 | 1.797E-02 | 2.794E-03 | T | C | 7.350E-01 |
| BB | rs7093444 | -9.607E-03 | 3.694E-03 | C | T | 1.275E-01 |
| BB | rs74717224 | -1.401E-02 | 5.245E-03 | T | G | 5.857E-02 |
| BB | rs10067003 | 8.417E-03 | 2.905E-03 | T | C | 2.422E-01 |
| BB | rs115412659 | 1.245E-02 | 5.290E-03 | A | G | 5.823E-02 |
| BB | rs11750184 | -6.937E-03 | 2.791E-03 | A | G | 2.665E-01 |
| BB | rs36079 | 6.912E-03 | 2.926E-03 | A | G | 2.312E-01 |
| BB | rs4705073 | 6.751E-03 | 2.473E-03 | C | T | 4.976E-01 |
| BB | rs7737361 | -1.413E-02 | 3.178E-03 | A | G | 1.913E-01 |
| BB | rs35866749 | -1.064E-02 | 3.403E-03 | G | A | 1.547E-01 |
| BB | rs144549900 | -1.300E-02 | 5.023E-03 | C | A | 6.621E-02 |
| BB | rs2052130 | 1.111E-02 | 3.512E-03 | A | G | 1.477E-01 |
| CCB | rs4765935 | 5.752E-03 | 2.709E-03 | C | A | 6.986E-01 |
| CCB | rs55909860 | 1.848E-02 | 6.971E-03 | T | C | 3.399E-02 |
| CCB | rs882193 | 6.066E-03 | 2.579E-03 | A | G | 6.183E-01 |
| CCB | rs111969626 | -5.380E-03 | 2.468E-03 | A | G | 5.318E-01 |
| CCB | rs146992327 | 3.299E-02 | 1.209E-02 | C | T | 1.088E-02 |
| CCB | rs4583644 | -2.072E-02 | 7.039E-03 | A | G | 3.161E-02 |
| CCB | rs6445583 | 1.386E-02 | 2.823E-03 | A | G | 7.458E-01 |
| CCB | rs6797014 | 7.207E-03 | 2.551E-03 | T | C | 3.717E-01 |
| CCB | rs79020595 | -3.399E-02 | 1.006E-02 | A | G | 1.726E-02 |
| CCB | rs118166304 | -1.938E-02 | 9.370E-03 | G | T | 1.766E-02 |
| CCB | rs198535 | -1.102E-02 | 2.506E-03 | G | A | 4.177E-01 |
| CCB | rs34157595 | -5.087E-03 | 2.532E-03 | C | T | 3.950E-01 |
| CCB | rs8065903 | -7.571E-03 | 2.795E-03 | G | A | 7.381E-01 |
| CCB | rs117177120 | 1.809E-02 | 5.430E-03 | G | T | 5.730E-02 |
| CCB | rs12926678 | -5.138E-03 | 2.575E-03 | T | C | 3.830E-01 |
| CCB | rs1977100 | -6.019E-03 | 2.638E-03 | G | A | 3.203E-01 |
| CCB | rs4347630 | 7.868E-03 | 3.295E-03 | C | T | 8.154E-01 |
| CCB | rs71380229 | 1.042E-02 | 4.584E-03 | C | A | 7.912E-02 |
| CCB | rs8053994 | -6.127E-03 | 2.537E-03 | A | G | 5.550E-01 |
| CCB | rs117741951 | -1.823E-02 | 7.466E-03 | T | C | 2.940E-02 |
| CCB | rs136832 | 6.252E-03 | 3.158E-03 | T | C | 1.864E-01 |
| CCB | rs76492099 | -1.191E-02 | 5.094E-03 | A | G | 6.301E-02 |
| CCB | rs34484573 | -1.972E-02 | 3.707E-03 | A | G | 1.261E-01 |
| CCB | rs3806708 | -8.168E-03 | 3.821E-03 | C | T | 1.178E-01 |
| CCB | rs73077175 | -1.122E-02 | 2.611E-03 | A | G | 3.331E-01 |
| CCB | rs74471041 | 6.897E-03 | 2.806E-03 | G | T | 2.588E-01 |
| CCB | rs2338115 | -5.892E-03 | 2.476E-03 | T | C | 5.544E-01 |
| CCB | rs61554907 | 8.199E-03 | 3.974E-03 | T | G | 1.095E-01 |
| CCB | rs71369724 | 1.183E-02 | 5.465E-03 | A | G | 5.401E-02 |
| CCB | rs10764322 | 1.383E-02 | 2.665E-03 | G | A | 3.107E-01 |
| CCB | rs10828906 | 1.122E-02 | 2.791E-03 | T | C | 7.351E-01 |
| CCB | rs115101482 | 3.077E-02 | 1.060E-02 | A | G | 1.379E-02 |
| CCB | rs117926523 | 2.921E-02 | 1.016E-02 | G | A | 1.589E-02 |
| CCB | rs12416030 | 1.098E-02 | 3.106E-03 | C | T | 2.017E-01 |
| CCB | rs140766681 | 2.171E-02 | 8.551E-03 | C | A | 2.116E-02 |
| CCB | rs1757234 | -7.722E-03 | 3.844E-03 | A | G | 1.164E-01 |
| CCB | rs184067968 | -2.449E-02 | 1.007E-02 | A | C | 1.646E-02 |
| CCB | rs2255266 | -7.116E-03 | 3.047E-03 | C | T | 7.938E-01 |
| CCB | rs2488158 | 6.929E-03 | 3.314E-03 | A | G | 1.660E-01 |
| CCB | rs2488161 | 8.202E-03 | 3.537E-03 | G | A | 1.611E-01 |
| CCB | rs7922241 | 1.126E-02 | 3.044E-03 | G | A | 7.915E-01 |
| CCB | rs12317778 | -2.122E-02 | 4.493E-03 | C | T | 8.226E-02 |
| CCB | rs2453467 | 6.459E-03 | 3.034E-03 | T | C | 7.883E-01 |
| CCB | rs115904908 | -3.907E-02 | 1.055E-02 | T | C | 1.376E-02 |
| CCB | rs2139419 | 7.193E-03 | 2.577E-03 | T | C | 6.420E-01 |
| CCB | rs4254462 | -7.479E-03 | 3.446E-03 | C | T | 1.525E-01 |
| CCB | rs78784512 | 1.698E-02 | 8.618E-03 | T | C | 2.284E-02 |
| CCB | rs11605129 | -1.910E-02 | 5.508E-03 | T | C | 5.276E-02 |
| CCB | rs117842198 | -2.650E-02 | 1.266E-02 | A | G | 1.073E-02 |
| CCB | rs7112615 | 1.194E-02 | 5.123E-03 | A | C | 9.384E-01 |
| CCB | rs7129324 | -2.358E-02 | 1.024E-02 | A | G | 1.664E-02 |
| CCB | rs12145535 | 7.678E-03 | 2.618E-03 | T | C | 3.294E-01 |
| HMGCR | rs721901 | 5.055E-02 | 8.380E-03 | T | C | 3.508E-01 |
| HMGCR | rs11949107 | 7.163E-02 | 8.793E-03 | A | G | 2.865E-01 |
| HMGCR | rs142704706 | 1.298E-01 | 1.885E-02 | T | C | 5.236E-02 |
| HMGCR | rs6453133 | 1.278E-01 | 8.537E-03 | G | A | 3.130E-01 |
| HMGCR | rs144070522 | 2.599E-01 | 2.889E-02 | C | T | 2.210E-02 |
| HMGCR | rs11956764 | 1.013E-01 | 1.816E-02 | C | T | 5.528E-02 |
| HMGCR | rs55810502 | 6.896E-02 | 9.921E-03 | G | A | 2.001E-01 |
| PCSK9 | rs4927191 | -1.817E-01 | 2.681E-02 | C | T | 3.097E-01 |
| PCSK9 | rs693668 | 1.874E-01 | 2.453E-02 | A | G | 3.888E-01 |

SNP, single-nucleotide polymorphism; SE, standard error; EA, effect allele; OA, other allele; EAF, effect allele frequency; BBs, beta-blockers; CCBs, calcium channel blockers.
