# Supplementary material for: Association of genetic variants related to combined lipid-lowering and antihypertensive therapies with risk of cardiovascular disease: 2 × 2 factorial Mendelian randomization analyses
Source: BMC Med. 2024 May 20;22:201. doi: 10.1186/s12916-024-03407-x (PMC11103938; doi:10.1186/s12916-024-03407-x)
Supplement: Supplementary file 2 — Additional file 2: Fig. S1. Participant flow chart of the study in the UK Biobank. Fig. S2. Associations of exposure to genetically proxied lipid-lowering drugs, genetically proxied antihypertensive drugs, or both with risk of ischemic stroke. Fig. S3. Associations of exposure to genetically proxied lipid-lowering drugs, genetically proxied antihypertensive drugs, or both with risk of hemorrhagic stroke. Fig. S4. Associations of exposure to genetically proxied lipid-lowering drugs, genetically proxied antihypertensive drugs, or both with risk of heart failure. Fig. S5. Associations of exposure to genetically proxied lipid-lowering drugs, genetically proxied antihypertensive drugs, or both with risk of diabetes mellitus. Fig. S6. Associations of exposure to genetically proxied lipid-lowering drugs, genetically proxied antihypertensive drugs, or both with risk of chronic kidney disease. Fig. S7. Associations of exposure to genetically proxied lipid-lowering drugs, genetically proxied antihypertensive drugs, or both with risk of dementia. Fig. S8. Associations of exposure to genetically proxied lipid-lowering drugs, genetically proxied antihypertensive drugs, or both with risk of peripheral artery diseases. Fig. S9. Associations of exposure to genetically proxied lipid-lowering drugs, genetically proxied antihypertensive drugs, or both with risk of cardiovascular diseases, with additional adjustment for body mass index. Fig. S10. Associations of exposure to genetically proxied lipid-lowering drugs, genetically proxied antihypertensive drugs, or both with risk of coronary artery diseases, with additional adjustment for body mass index. Fig. S11. Associations of exposure to genetically proxied lipid-lowering drugs, genetically proxied antihypertensive drugs, or both with risk of stroke, with additional adjustment for body mass index. Fig. S12. Associations of exposure to genetically proxied lipid-lowering drugs, genetically proxied antihypertensive drugs, or both with ri [file 12916_2024_3407_MOESM2_ESM.docx]

# Additional file2


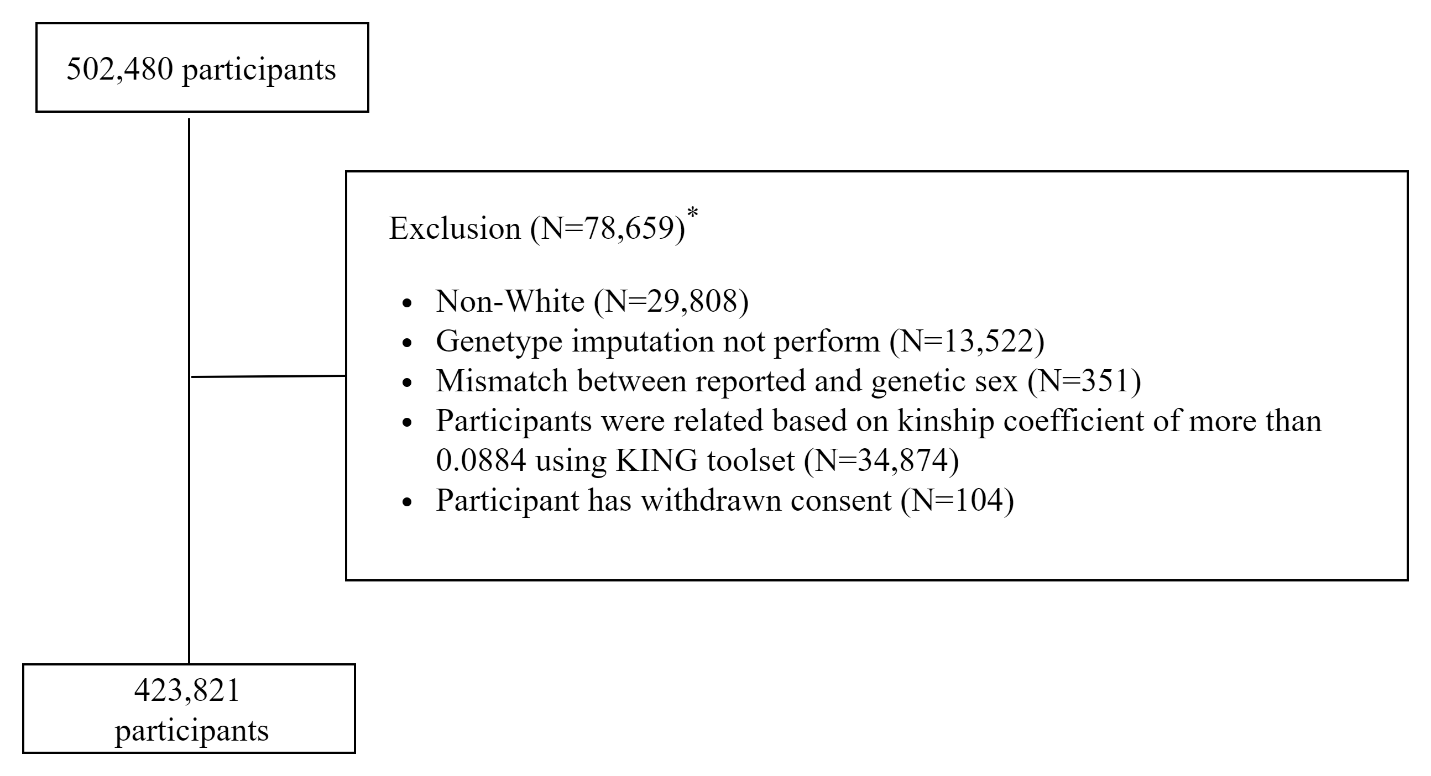


**Fig. S1**. **Participant flow chart of the study in the UK Biobank.**

*Exclusion was done in a sequential order.

**Fig. S2. Associations of exposure to genetically proxied lipid-lowering drugs, genetically proxied antihypertensive drugs, or both with risk of ischemic stroke.**

BBs, beta-blockers; CCBs, calcium channel blockers.

Notes: The 2×2 analysis divided participants into 4 groups according to the median genetic scores of lipid-lowering drugs and antihypertensive drugs: “Both scores ≥ median” represented the group of placebos; “statin/PCSK9 score < median” was the proxy for statin or PCSK9 inhibitors use; “BBs/CCBs score < median” was the proxy for BBs or CCBs medication; “both scores < median” was the proxy for combined medications. Continuous analysis refers to the associations where the 2 genetic scores were included on a continuous scale (per SD decrease) as well as the interaction between the 2 scores.

**Fig. S3. Associations of exposure to genetically proxied lipid-lowering drugs, genetically proxied antihypertensive drugs, or both with risk of hemorrhagic stroke.**

BBs, beta-blockers; CCBs, calcium channel blockers.

Notes: The 2×2 analysis divided participants into 4 groups according to the median genetic scores of lipid-lowering drugs and antihypertensive drugs: “Both scores ≥ median” represented the group of placebos; “statin/PCSK9 score < median” was the proxy for statin or PCSK9 inhibitors use; “BBs/CCBs score < median” was the proxy for BBs or CCBs medication; “both scores < median” was the proxy for combined medications. Continuous analysis refers to the associations where the 2 genetic scores were included on a continuous scale (per SD decrease) as well as the interaction between the 2 scores.

**Fig. S4. Associations of exposure to genetically proxied lipid-lowering drugs, genetically proxied antihypertensive drugs, or both with risk of heart failure.**

BBs, beta-blockers; CCBs, calcium channel blockers.

Notes: The 2×2 analysis divided participants into 4 groups according to the median genetic scores of lipid-lowering drugs and antihypertensive drugs: “Both scores ≥ median” represented the group of placebos; “statin/PCSK9 score < median” was the proxy for statin or PCSK9 inhibitors use; “BBs/CCBs score < median” was the proxy for BBs or CCBs medication; “both scores < median” was the proxy for combined medications. Continuous analysis refers to the associations where the 2 genetic scores were included on a continuous scale (per SD decrease) as well as the interaction between the 2 scores.

**Fig. S5. Associations of exposure to genetically proxied lipid-lowering drugs, genetically proxied antihypertensive drugs, or both with risk of diabetes mellitus.**

Notes: The 2×2 analysis divided participants into 4 groups according to the median genetic scores of lipid-lowering drugs and antihypertensive drugs: “Both scores ≥ median” represented the group of placebos; “statin/PCSK9 score < median” was the proxy for statin or PCSK9 inhibitors use; “BBs/CCBs score < median” was the proxy for BBs or CCBs medication; “both scores < median” was the proxy for combined medications. Continuous analysis refers to the associations where the 2 genetic scores were included on a continuous scale (per SD decrease) as well as the interaction between the 2 scores.

**Fig. S6. Associations of exposure to genetically proxied lipid-lowering drugs, genetically proxied antihypertensive drugs, or both with risk of chronic kidney disease.**

Notes: The 2×2 analysis divided participants into 4 groups according to the median genetic scores of lipid-lowering drugs and antihypertensive drugs: “Both scores ≥ median” represented the group of placebos; “statin/PCSK9 score < median” was the proxy for statin or PCSK9 inhibitors use; “BBs/CCBs score < median” was the proxy for BBs or CCBs medication; “both scores < median” was the proxy for combined medications. Continuous analysis refers to the associations where the 2 genetic scores were included on a continuous scale (per SD decrease) as well as the interaction between the 2 scores.

**Fig. S7. Associations of exposure to genetically proxied lipid-lowering drugs, genetically proxied antihypertensive drugs, or both with risk of dementia.**

Notes: The 2×2 analysis divided participants into 4 groups according to the median genetic scores of lipid-lowering drugs and antihypertensive drugs: “Both scores ≥ median” represented the group of placebos; “statin/PCSK9 score < median” was the proxy for statin or PCSK9 inhibitors use; “BBs/CCBs score < median” was the proxy for BBs or CCBs medication; “both scores < median” was the proxy for combined medications. Continuous analysis refers to the associations where the 2 genetic scores were included on a continuous scale (per SD decrease) as well as the interaction between the 2 scores.

**Fig. S8. Associations of exposure to genetically proxied lipid-lowering drugs, genetically proxied antihypertensive drugs, or both with risk of peripheral artery diseases.**

Notes: The 2×2 analysis divided participants into 4 groups according to the median genetic scores of lipid-lowering drugs and antihypertensive drugs: “Both scores ≥ median” represented the group of placebos; “statin/PCSK9 score < median” was the proxy for statin or PCSK9 inhibitors use; “BBs/CCBs score < median” was the proxy for BBs or CCBs medication; “both scores < median” was the proxy for combined medications. Continuous analysis refers to the associations where the 2 genetic scores were included on a continuous scale (per SD decrease) as well as the interaction between the 2 scores.

## Sensitivity analyses

 **Fig. S9. Associations of exposure to genetically proxied lipid-lowering drugs, genetically proxied antihypertensive drugs, or both with risk of cardiovascular diseases, with additional adjustment for body mass index.**

Notes: The 2×2 analysis divided participants into 4 groups according to the median genetic scores of lipid-lowering drugs and antihypertensive drugs: “Both scores ≥ median” represented the group of placebos; “statin/PCSK9 score < median” was the proxy for statin or PCSK9 inhibitors use; “BBs/CCBs score < median” was the proxy for BBs or CCBs medication; “both scores < median” was the proxy for combined medications. Continuous analysis refers to the associations where the 2 genetic scores were included on a continuous scale (per SD decrease) as well as the interaction between the 2 scores.

 **Fig. S10. Associations of exposure to genetically proxied lipid-lowering drugs, genetically proxied antihypertensive drugs, or both with risk of coronary artery diseases, with additional adjustment for body mass index.**

Notes: The 2×2 analysis divided participants into 4 groups according to the median genetic scores of lipid-lowering drugs and antihypertensive drugs: “Both scores ≥ median” represented the group of placebos; “statin/PCSK9 score < median” was the proxy for statin or PCSK9 inhibitors use; “BBs/CCBs score < median” was the proxy for BBs or CCBs medication; “both scores < median” was the proxy for combined medications. Continuous analysis refers to the associations where the 2 genetic scores were included on a continuous scale (per SD decrease) as well as the interaction between the 2 scores.

 **Fig. S11. Associations of exposure to genetically proxied lipid-lowering drugs, genetically proxied antihypertensive drugs, or both with risk of stroke, with additional adjustment for body mass index.**

Notes: The 2×2 analysis divided participants into 4 groups according to the median genetic scores of lipid-lowering drugs and antihypertensive drugs: “Both scores ≥ median” represented the group of placebos; “statin/PCSK9 score < median” was the proxy for statin or PCSK9 inhibitors use; “BBs/CCBs score < median” was the proxy for BBs or CCBs medication; “both scores < median” was the proxy for combined medications. Continuous analysis refers to the associations where the 2 genetic scores were included on a continuous scale (per SD decrease) as well as the interaction between the 2 scores. **Fig. S12. Associations of exposure to genetically proxied lipid-lowering drugs, genetically proxied antihypertensive drugs, or both with risk of cardiovascular disease, as defined by a kinship coefficient of 0.0442.**

Notes: The 2×2 analysis divided participants into 4 groups according to the median genetic scores of lipid-lowering drugs and antihypertensive drugs: “Both scores ≥ median” represented the group of placebos; “statin/PCSK9 score < median” was the proxy for statin or PCSK9 inhibitors use; “BBs/CCBs score < median” was the proxy for BBs or CCBs medication; “both scores < median” was the proxy for combined medications. Continuous analysis refers to the associations where the 2 genetic scores were included on a continuous scale (per SD decrease) as well as the interaction between the 2 scores. **Fig. S13. Associations of exposure to genetically proxied lipid-lowering drugs, genetically proxied antihypertensive drugs, or both with risk of coronary artery disease, as defined by a kinship coefficient of 0.0442.**

Notes: The 2×2 analysis divided participants into 4 groups according to the median genetic scores of lipid-lowering drugs and antihypertensive drugs: “Both scores ≥ median” represented the group of placebos; “statin/PCSK9 score < median” was the proxy for statin or PCSK9 inhibitors use; “BBs/CCBs score < median” was the proxy for BBs or CCBs medication; “both scores < median” was the proxy for combined medications. Continuous analysis refers to the associations where the 2 genetic scores were included on a continuous scale (per SD decrease) as well as the interaction between the 2 scores. **Fig. S14. Associations of exposure to genetically proxied lipid-lowering drugs, genetically proxied antihypertensive drugs, or both with risk of stroke, as defined by a kinship coefficient of 0.0442.**

Notes: The 2×2 analysis divided participants into 4 groups according to the median genetic scores of lipid-lowering drugs and antihypertensive drugs: “Both scores ≥ median” represented the group of placebos; “statin/PCSK9 score < median” was the proxy for statin or PCSK9 inhibitors use; “BBs/CCBs score < median” was the proxy for BBs or CCBs medication; “both scores < median” was the proxy for combined medications. Continuous analysis refers to the associations where the 2 genetic scores were included on a continuous scale (per SD decrease) as well as the interaction between the 2 scores. **Fig. S15. Associations of exposure to genetically proxied lipid-lowering drugs, genetically proxied antihypertensive drugs, or both with risk of cardiovascular disease, using 30th percentiles and 70th percentile for grouping.**

Notes: The 2×2 analysis divided participants into 4 groups according to the median genetic scores of lipid-lowering drugs and antihypertensive drugs: “Both scores ≥ 70th percentile” represented the group of placebos; “statin/PCSK9 score < 30th percentile” was the proxy for statin or PCSK9 inhibitors use; “BBs/CCBs score < 30th percentile” was the proxy for BBs or CCBs medication; “both scores < 30th percentile” was the proxy for combined medications. Continuous analysis refers to the associations where the 2 genetic scores were included on a continuous scale (per SD decrease) as well as the interaction between the 2 scores. **Fig. S16. Associations of exposure to genetically proxied lipid-lowering drugs, genetically proxied antihypertensive drugs, or both with risk of coronary artery disease, using 30th percentiles and 70th percentile for grouping.**

Notes: The 2×2 analysis divided participants into 4 groups according to the median genetic scores of lipid-lowering drugs and antihypertensive drugs: “Both scores ≥ 70th percentile” represented the group of placebos; “statin/PCSK9 score < 30th percentile” was the proxy for statin or PCSK9 inhibitors use; “BBs/CCBs score < 30th percentile” was the proxy for BBs or CCBs medication; “both scores < 30th percentile” was the proxy for combined medications. Continuous analysis refers to the associations where the 2 genetic scores were included on a continuous scale (per SD decrease) as well as the interaction between the 2 scores. **Fig. S17. Associations of exposure to genetically proxied lipid-lowering drugs, genetically proxied antihypertensive drugs, or both with risk of stroke, using 30th percentiles and 70th percentile for grouping.**

Notes: The 2×2 analysis divided participants into 4 groups according to the median genetic scores of lipid-lowering drugs and antihypertensive drugs: “Both scores ≥ 70th percentile” represented the group of placebos; “statin/PCSK9 score < 30th percentile” was the proxy for statin or PCSK9 inhibitors use; “BBs/CCBs score < 30th percentile” was the proxy for BBs or CCBs medication; “both scores < 30th percentile” was the proxy for combined medications. Continuous analysis refers to the associations where the 2 genetic scores were included on a continuous scale (per SD decrease) as well as the interaction between the 2 scores. **Fig. S18. Associations of exposure to genetically proxied lipid-lowering drugs, genetically proxied antihypertensive drugs, or both with risk of cardiovascular diseases, with eQTL as the genetic proxies for these four drugs.**

Notes: The 2×2 analysis divided participants into 4 groups according to the median genetic scores of lipid-lowering drugs and antihypertensive drugs: “Both scores ≥ median” represented the group of placebos; “statin/PCSK9 score < median” was the proxy for statin or PCSK9 inhibitors use; “BBs/CCBs score < median” was the proxy for BBs or CCBs medication; “both scores < median” was the proxy for combined medications. Continuous analysis refers to the associations where the 2 genetic scores were included on a continuous scale (per SD decrease) as well as the interaction between the 2 scores.

 **Fig. S19. Associations of exposure to genetically proxied lipid-lowering drugs, genetically proxied antihypertensive drugs, or both with risk of coronary artery disease, with eQTL as the genetic proxies for these four drugs.**

Notes: The 2×2 analysis divided participants into 4 groups according to the median genetic scores of lipid-lowering drugs and antihypertensive drugs: “Both scores ≥ median” represented the group of placebos; “statin/PCSK9 score < median” was the proxy for statin or PCSK9 inhibitors use; “BBs/CCBs score < median” was the proxy for BBs or CCBs medication; “both scores < median” was the proxy for combined medications. Continuous analysis refers to the associations where the 2 genetic scores were included on a continuous scale (per SD decrease) as well as the interaction between the 2 scores.

 **Fig. S20. Associations of exposure to genetically proxied lipid-lowering drugs, genetically proxied antihypertensive drugs, or both with risk of stroke, with eQTL as the genetic proxies for these four drugs.**

Notes: The 2×2 analysis divided participants into 4 groups according to the median genetic scores of lipid-lowering drugs and antihypertensive drugs: “Both scores ≥ median” represented the group of placebos; “statin/PCSK9 score < median” was the proxy for statin or PCSK9 inhibitors use; “BBs/CCBs score < median” was the proxy for BBs or CCBs medication; “both scores < median” was the proxy for combined medications. Continuous analysis refers to the associations where the 2 genetic scores were included on a continuous scale (per SD decrease) as well as the interaction between the 2 scores.

**Fig. S21. Associations of exposure to genetically proxied lipid-lowering drugs, genetically proxied antihypertensive drugs, or both with risk of cardiovascular disease, excluding participants who used lipid-lowering or antihypertensive drugs at baseline.**

Notes: The 2×2 analysis divided participants into 4 groups according to the median genetic scores of lipid-lowering drugs and antihypertensive drugs: “Both scores ≥ median” represented the group of placebos; “statin/PCSK9 score < median” was the proxy for statin or PCSK9 inhibitors use; “BBs/CCBs score < median” was the proxy for BBs or CCBs medication; “both scores < median” was the proxy for combined medications. Continuous analysis refers to the associations where the 2 genetic scores were included on a continuous scale (per SD decrease) as well as the interaction between the 2 scores.

**Fig. S22. Associations of exposure to genetically proxied lipid-lowering drugs, genetically proxied antihypertensive drugs, or both with risk of coronary artery disease, excluding participants who used lipid-lowering or antihypertensive drugs at baseline.**

Notes: The 2×2 analysis divided participants into 4 groups according to the median genetic scores of lipid-lowering drugs and antihypertensive drugs: “Both scores ≥ median” represented the group of placebos; “statin/PCSK9 score < median” was the proxy for statin or PCSK9 inhibitors use; “BBs/CCBs score < median” was the proxy for BBs or CCBs medication; “both scores < median” was the proxy for combined medications. Continuous analysis refers to the associations where the 2 genetic scores were included on a continuous scale (per SD decrease) as well as the interaction between the 2 scores.

**Fig. S23. Associations of exposure to genetically proxied lipid-lowering drugs, genetically proxied antihypertensive drugs, or both with risk of stroke, excluding participants who used lipid-lowering or antihypertensive drugs at baseline.**

Notes: The 2×2 analysis divided participants into 4 groups according to the median genetic scores of lipid-lowering drugs and antihypertensive drugs: “Both scores ≥ median” represented the group of placebos; “statin/PCSK9 score < median” was the proxy for statin or PCSK9 inhibitors use; “BBs/CCBs score < median” was the proxy for BBs or CCBs medication; “both scores < median” was the proxy for combined medications. Continuous analysis refers to the associations where the 2 genetic scores were included on a continuous scale (per SD decrease) as well as the interaction between the 2 scores.
